# Clinical impact of testing for mutations and microRNAs in thyroid nodules

**DOI:** 10.1002/dc.24190

**Published:** 2019-04-23

**Authors:** John Woody Sistrunk, Alexander Shifrin, Marc Frager, Ricardo H. Bardales, Johnson Thomas, Norman Fishman, Philip Goldberg, Richard Guttler, Edward Grant

**Affiliations:** ^1^ Jackson Thyroid & Endocrine Clinic Jackson Mississippi; ^2^ Department of Surgery, Jersey Shore University Medical Center, Neptune New Jersey; ^3^ East Coast Medical Associates Boca Raton Florida; ^4^ Precision Pathology/Outpatient Pathology Associates Sacramento California; ^5^ Mercy Clinic Endocrinology Springfield Missouri; ^6^ Diabetes & Endocrinology Specialists Chesterfield Missouri; ^7^ Endocrine Associates of Connecticut Branford Connecticut; ^8^ Thyroid Center of Santa Monica Santa Monica California; ^9^ Department of Radiology, University of Southern California, Keck School of Medicine Los Angeles California

**Keywords:** microRNA classifier, molecular analysis, mutation, patient outcomes, thyroid nodules

## Abstract

**Background:**

We report results of a multicenter clinical experience study examining the likelihood of patients with indeterminate thyroid nodules to undergo surgery or have malignant outcome based on multiplatform combination mutation and microRNA testing (MPT).

**Methods:**

MPT assessed mutations in BRAF, HRAS, KRAS, NRAS, and PIK3CA genes, PAX8/PPARγ, RET/PTC1, and RET/PTC3 gene rearrangements, and the expression of 10 microRNAs. Baseline clinical information at the time of MPT and clinical follow‐up records were reviewed for 337 patients, of which 80% had negative MPT results. Kaplan Meier analysis for cumulative probability of survival without having a surgical procedure or malignant diagnosis over the course of patient follow‐up was determined for MPT results of 180 patients, among which only 14% had malignancy.

**Results:**

A negative MPT result in nodules with Bethesda III or IV cytology (2009) conferred a high probability of non‐surgical treatment, with only 11% expected to undergo surgery and a high probability of survival without malignancy (92%) for up to 2 years follow up. A positive MPT result conferred a 57% probability of malignancy and was an independent risk factor for undergoing surgical treatment (Hazard Ratio [HR] 9.2, 95% confidence intervals 5.4‐15.9, *P* < .0001) and for malignancy (HR 13.4, 95% confidence intervals 4.8‐37.2, *P* < .0001). For nodules with weak driver mutations, positive microRNA test results supported high risk of cancer while negative results downgraded cancer risk.

**Conclusion:**

MPT results are predictive of real‐world decisions to surgically treat indeterminate thyroid nodules, with those decisions being appropriately aligned with a patient's risk of malignancy over time.

## 1 INTRODUCTION

When managing thyroid nodules physicians need to distinguish potentially malignant nodules from those with benign disease to limit unnecessary surgery. Ultrasound imaging followed by cytopathology review of fine needle aspirations (FNA) is the current standard of care for the diagnosis of thyroid nodule malignancy. Although cytopathology categorizes the majority of nodules as benign or malignant with a high degree of certainty, up to 30% of nodules are reported as indeterminate, where the presence of malignancy is less certain.^1^, ^2^, ^3^, ^4^ Only 12%‐20% of surgically resected indeterminate nodules that have atypia of undetermined significance or a follicular lesion of undetermined significance (AUS/FLUS, Bethesda category III) diagnosis will have malignancy.^5^ The malignancy rate only increases to 25%‐33% in nodules that are a follicular neoplasm or are suspicious for follicular neoplasm (FN/SFN, Bethesda category IV).^5^ Rates of malignancy in Bethesda category III and IV nodules are likely even lower when nodules that undergo observation rather than surgery are also considered. Regardless, indeterminate cytopathology often triggers unnecessary diagnostic lobectomy and even total thyroidectomy, given concern for cancer.

Molecular testing of nodules with indeterminate cytology diagnosis following a thyroid nodule FNA has been incorporated into routine care to help resolve diagnostic uncertainty.^6^ While mutations strongly associated with malignancy, such as BRAF V600E, RET fusions, and TERT can assist in surgical decision making, other mutations considered weak drivers of cancer carry less certainty.^7^, ^8^, ^9^, ^10^ RAS mutations, the most common type in thyroid nodules, have been reported to have variable positive and negative predictive value for malignancy, often occurring in benign nodules.^11^, ^12^, ^13^, ^14^, ^15^ Furthermore, residual risk of malignancy (5%‐25%) is present in patients who lack any detectable mutational change.^16^, ^17^

Multiple studies have described the ability of RNA‐based risk classifiers to help resolve diagnostic uncertainty in indeterminate thyroid nodules.^18^, ^19^, ^20^ A messenger RNA risk classifier can help to rule out the need for surgery in indeterminate nodules due to its high negative predictive value for malignancy.^19^, ^20^ However, its less than optimal positive predictive value limits the ability to rule in the need for surgery, especially in AUS/FLUS nodules, where malignancy rates are low.^19^, ^20^, ^21^ Comparatively, non‐coding microRNA based risk classifiers have shown similarly high negative predictive value (NPV) for malignancy and superior positive predictive value when used in combination with analysis for oncogenic mutations and messenger RNA fusion transcripts.^18^ Given the improved performance of the combination approach, this multiplatform combination of mutations and microRNA test (MPT) can effectively help to rule in and rule out the need for surgery. Clinical experience has shown that microRNA analysis can also help reclassify cancer risk under variable mutational profiles, including when patients have weak driver mutations such as RAS.^16^ However, there has previously been limited data supporting the impact of MPT results on real‐world decisions to surgically treat nodules or on the outcomes of patients who have undergone such testing in clinical practice.

Herein, we report the results of a multicenter clinical experience study of nodules that underwent MPT testing in clinical practice. We evaluated the impact of MPT results on real‐world decisions to surgically treat indeterminate thyroid nodules with Bethesda category III or IV diagnosis. We also determined if those surgical decisions were appropriately aligned with a patient's risk of malignancy over a period of time based on MPT results.

## 2 METHODS

### 2.1 Study design and patient population

The study was an observational clinical experience study of consecutive patients who had multiplatform combination mutation analysis (ThyGenX) and microRNA classification (ThyraMIR) testing prescribed by their physician as standard of care. The study included medical record review, was non‐interventional and was reviewed and approved by a central independent ethics review board (Quorum IRB #32262). Informed consent was waived by the IRB due to minimal risk. The study included nine participating sites from across the United States. Subjects included patients ≥18 years of age who had initial, baseline molecular testing performed between July 2015 and January 2018 for indeterminate thyroid nodules due to Bethesda III or IV category cytology and who did not have a history of thyroid cancer. Study exclusion criteria included any subject that did not have past MPT results and those that had incidental thyroid cancer findings unrelated to their tested thyroid nodule.

### 2.2 Baseline patient characteristics

Baseline patient demographic and clinical information was abstracted from de‐identified patient history and physical examination records, ultrasound, cytology records, and clinical notes. This information included the patient age, nodule size, laboratory values at the time of molecular testing, family and personal histories of cancer (thyroid and non‐thyroid), and non‐malignant thyroid disease. Baseline information reflected that which was present when molecular testing was prescribed. Cytology results and corresponding Bethesda categorization (2009) were derived from cytology records of fine needle aspirates (FNA) for which molecular testing was prescribed.

### 2.3 Baseline multiplatform mutation and microRNA testing (MPT)

Multiplatform mutation and microRNA testing (MPT) was performed clinically on thyroid nodule FNA aspirates as part of standard of care using ThyGenX and ThyraMIR commercial tests (Interpace Diagnostics). Targeted next‐generation sequencing (NGS) analysis was used to detect mutations in BRAF, HRAS, KRAS, NRAS, and PIK3CA genes, and oncogenic messenger RNA fusion transcripts for PAX8/PPARγ, NCOA4/RET (RET/PTC1), and CCDC6/RET (RET/PTC3) rearrangements as previously described.^18^ microRNA expression analysis was performed by reverse transcription quantitative PCR for miR‐29b‐1‐5p, miR‐31‐5p, miR‐138‐1‐3p, miR‐139‐5p, miR‐146b‐5p, miR‐155, miR‐204‐5p, miR‐222‐3p, miR‐375, and miR‐551b‐3p as previously described.^18^

MPT results were abstracted from patient medical reports and categorized as positive or negative using two approaches. The first approach was as previously described,^18^ where a positive MPT result was defined by the presence of any mutation and/or the presence of a positive microRNA test result. A negative MPT result was defined by the lack of all detectable mutational change in combination with a negative microRNA test result. The second approach defined a positive MPT result by the presence of strong driver mutations (ie, BRAFV600E mutation or RET fusions) and/or the presence of a positive microRNA test result. A negative MPT result was defined by a negative microRNA test result in combination with either a lack of detectable mutational change or the presence of weak driver mutations (ie, RAS, PAX8/PPARγ fusion, PIK3CA, uncommon BRAF K601E)**.**

### 2.4 Follow‐up records and patient outcomes

Patient follow‐up records included ultrasound, cytology, clinical notes, operative notes, and surgical pathology reports. The result of the last office visit or surgical pathology result was used to determine patient malignant events, with the subsequent date of this final visit recorded. Malignant events were defined by the presence of carcinoma in surgical pathology records (papillary thyroid carcinoma, follicular carcinoma, Hürthle cell carcinoma) or follow‐up repeat FNA malignant cytology results (Bethesda category VI). All other follow‐up results of patients were considered non‐malignant.

### 2.5 Statistical analysis

Statistical analysis was performed using R statistical software (r-project.org). *P*‐values <.05 were considered statistically significant. Malignancy‐free and surgery‐free survival was calculated from the date of molecular testing using Kaplan‐Meier survival analysis. A univariate and multivariate Cox proportional hazards regression model was used to assess the relationship between the independent clinical factors and MPT results compared to surgical treatment decisions and patient outcomes. The log rank test was used to compare the survival curves when appropriate. The expected absolute risk of undergoing surgery over a range of pretest surgery probabilities was calculated for MPT results using rates observed in the study cohort and Bayes theorem. The probability of a negative MPT result (ie, the rate of negative test results) was calculated based on the performance characteristics of the test in the study cohort over a range of various baseline malignancy rates.^22^

## 3 RESULTS

Our clinical experience study included 337 consecutive patients with AUS/FLUS (Bethesda category III) or FN/SFN (Bethesda Category IV) nodules from nine participating institutions that were prescribed MPT testing as part of their current standard of care. In our initial analysis, 80% (270/337) of study subjects had a negative MPT result as previously defined,^18^ that is, the absence of detectable mutational change in combination with a negative microRNA test result. Of the 337 patients, 157 did not have existing clinical follow‐up records following MPT testing. Nearly all of such cases (93%) had a negative MPT result. In total, 24% (81/337) of patients had surgical pathology records and 29% (99/337) had existing clinical follow‐up records at the end of the study period, resulting in a study cohort of 180/337 patients.

Baseline and follow‐up characteristics of the study cohort are shown in Table 1. At baseline, thyroid nodules were on average 2.1 cm in size, with a majority (69%) having Bethesda III cytology. Nearly half of all patients (48%) had a personal history of non‐malignant thyroid disease, 29% had a family history of non‐malignant thyroid disease, and 19% had a personal history of non‐thyroid cancer. Thyroid surgery occurred in 45% (81/180) of patients in the cohort, which was enriched for surgical events compared to the rate of thyroid surgery observed for all patients in the study (24% vs 45%, *P* = .0006). The median time to surgery was 2 months. More than half of the cohort (55%) had clinical follow‐up of at least 3 months and up to 2 years (median 9 months).

**Table 1:** Baseline and follow‐up characteristics of patients in the study cohort (N = 180)

Baseline			
Age	Median yrs (min‐max)	61	(19‐92)
Female	N (%)	125	(69%)
AUS/FLUS (Bethesda category III)	N (%)	124	(69%)
FN/SFN (Bethesda category IV)	N (%)	56	(31%)
Nodule size	Median cm (min‐max)	2.1	(0.6‐7.0)
Family history of thyroid cancer	N (%)	15	(8%)
Family history of non‐malignant thyroid disease	N (%)	53	(29%)
Personal history of non‐thyroid cancer	N (%)	34	(19%)
Personal history of thyroid disease	N (%)	86	(48%)
MPT negative results	N (%)	124	(69%)

Follow‐up			
Underwent surveillance	N (%)	99	(55%)
Clinical follow‐up time	Median mos (min‐max)	9	(3‐24)
Underwent surgery	N (%)	81	(45%)
Underwent total thyroidectomy	N (%)	37	(21%)
Underwent diagnostic lobectomy	N (%)	44	(24%)
Time to surgery	Median mos (min‐max)	2.0	(0‐19)
Malignant Diagnosis	N (%)	26	(14%)
Time to diagnosis of malignancy	Median mos (min‐max)	2.0	(0‐13)

Abbreviations: MPT, multiplatform mutation and microRNA test; mos, months; yrs, years; cm, centimeters; min, minimum; max, maximum.

**Figure 1:**
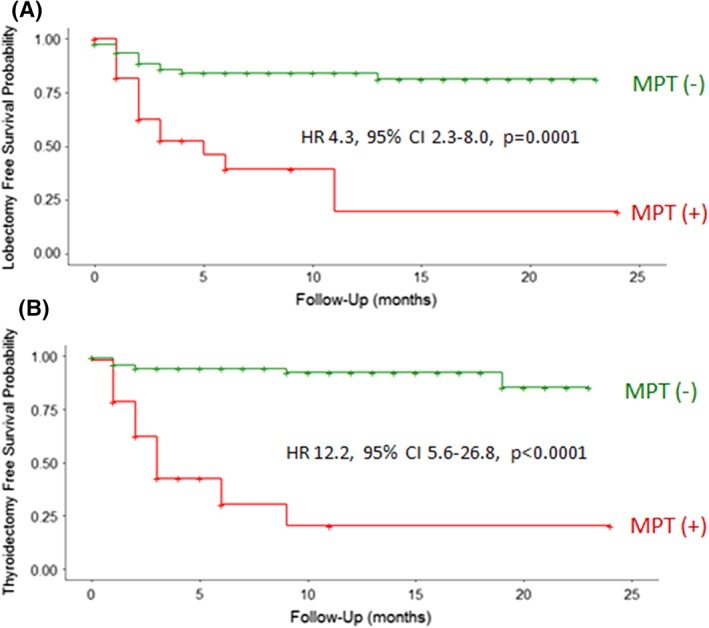
Kaplan Meier analysis for probability of survival without undergoing (A) diagnostic lobectomy or (B) total thyroidectomy surgery based on multiplatform mutation and microRNA (MPT) negative (green) or positive (red) results. Compared to patients with an MPT negative result, patients with an MPT positive result were at higher risk of undergoing a surgical procedure based on hazard ratio (HR) analysis. Abbreviation: CI, confidence interval

Over the 2‐year follow‐up period, we examined the probability at which patients underwent surgical treatment (diagnostic lobectomy or total thyroidectomy) for their thyroid nodules based on a positive or negative MPT result. Patients with a negative MPT result had a high probability of survival without undergoing a diagnostic lobectomy (81%) or total thyroidectomy (85%) surgery at 2 years follow‐up (Figure 1A,B). Comparatively, patients with a positive result were at higher risk of undergoing lobectomy (HR 4.3, 95%CI 2.3‐8.0, *P* = .0001) and even more so total thyroidectomy (HR 12.2, 95%CI 5.6‐26.8, *P* < .0001).

In univariate analysis, a positive MPT result (HR 7.94, 95% CI 4.93‐12.78, *P* < .0001) and to a lesser extent patient age (HR 0.98, 95%CI 0.96‐0.99, *P* = .0007) were the only significant risk factors for any type of surgical treatment (diagnostic lobectomy or total thyroidectomy; Table S1). In multivariate analysis, a positive MPT result remained a predictive risk factor for surgical treatment (HR 9.1, 95%CI 5.4‐15.2, *P* < .0001; Table S1). Both patient age (HR 0.98, 95%CI 0.96‐0.98, *P* = .0103) and nodule size (HR 1.03, 95%CI 1.01‐1.05, *P* = .0017) were also predictive risk factors for surgical treatment, but to a lesser extent than positive MPT results.

Given that our study cohort was enriched for surgical events, we examined the expected rate of surgery in patients with positive or negative MPT results over various baseline rates of surgery (Supplemental Figure 1). At a 24% surgery rate, which is the overall rate of surgery observed in the study, only 11% of patients with a negative MPT result were expected to undergo any type of surgical treatment (diagnostic lobectomy or total thyroidectomy) for their thyroid nodule. Comparatively, 84% of those with a positive result were expected to undergo surgical treatment.

**Figure 2:**
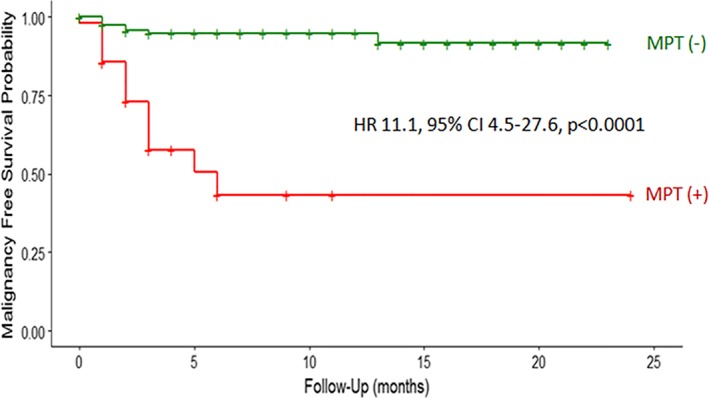
Kaplan Meier analysis for probability of survival without malignancy based on multiplatform mutation and microRNA (MPT) negative (green) or positive (red) results. Compared to patients with an MPT negative result, patients with an MPT positive result were at higher risk of malignancy by hazard ratio (HR) analysis. Abbreviation: CI, confidence interval

In addition to determining the impact of MPT results on surgical decisions, we examined whether those decisions were aligned with patient outcome. Over the 2 year follow‐up period, 14% (26/180) of patients had a surgical pathology determined malignant outcome with a 2 month average time to diagnosis of malignancy after MPT testing. Patients with a negative MPT result had a high probability (92%) of survival without malignancy for up to 2 years follow‐up (Figure 2). Comparatively, patients with a positive MPT result had a low probability (43%) of survival without malignancy and were at significantly higher risk of cancer (univariate HR 11.1, 95%CI 4.49‐27.63, *P* < .0001). When multivariate analysis was used to determine if baseline patient characteristics could be influencing risk of malignancy conferred by MPT results, a positive MPT result remained the only predictive risk factor for malignancy (HR 13.36, 95%CI 4.79‐37.22, *P* < .0001; Table S2). There were no other baseline patient characteristics that were predictive of malignancy in either univariate or multivariate analysis.

Because weak driver mutations are common in indeterminate nodules and have been shown to occur in both benign and malignant nodules,^11^, ^12^, ^13^, ^14^, ^15^ we examined the degree to which microRNA test results could alter risk stratification of nodules with weak drivers and impact the rate of a negative MPT result. For this analysis, a positive MPT result was defined by detection of strong driver mutations (ie, BRAFV600E mutation or RET fusions) and/or detection of a positive microRNA test result. A negative MPT result was defined by a negative microRNA test result in combination with either lack of detectable mutational change or the presence of weak driver mutations (ie, RAS, PAX8/PPARγ fusion, PIK3CA, uncommon BRAF K601E). In total, 92% (309/337) of patients who underwent MPT testing had a negative result in this secondary analysis.

**Figure 3:**
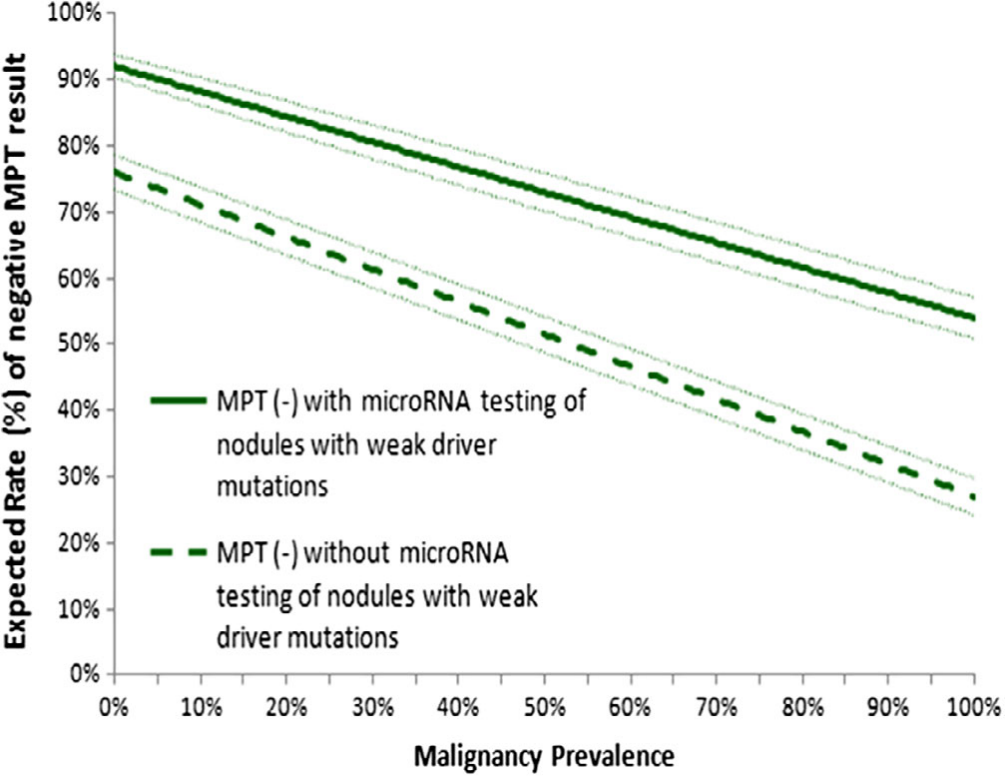
Sensitivity analysis for the expected rate of a negative multiplatform mutation and microRNA test (MPT) result based on incorporation of microRNA analysis of nodules with weak driver mutations (green solid line) into MPT or the lack there of (green dashed line) over various baseline rates of malignancy. Dotted lines represent 95% confidence intervals [Color figure can be viewed at wileyonlinelibrary.com]

Incorporation of microRNA testing of nodules with weak driver mutations into MPT significantly improved the rate of a negative MPT result from 69% (124/180) to 87% (156/180) in the study cohort. This improvement was consistent over various baseline rates of malignancy examined (Figure 3). When MPT included microRNA analysis of nodules with weak drivers, an 87% probability of malignancy‐free survival was observed for a negative diagnosis, and this carried no statistically different cancer risk than a negative MPT result without weak driver testing (87% vs 92%, *P* = .247). Positive results confirmed a low probability of malignancy‐free survival in the secondary analysis (38%), similar to the primary analysis (43%; 38% vs 43%, *P* = .363).

## 4 DISCUSSION

We performed a multicenter clinical experience study of Bethesda III and IV category nodules that underwent past clinical MPT testing to evaluate the impact of test results on future real‐world decisions to surgically treat indeterminate thyroid nodules. We also examined the risk of malignancy conferred by test results over time. Over the 2‐year follow‐up period, patients with a negative MPT result had a high probability of survival without undergoing a diagnostic lobectomy or total thyroidectomy and had a high probability of survival without malignancy. MPT positive results placed patients at higher risk of undergoing diagnostic lobectomy and even more so total thyroidectomy. A positive result was an independent risk factor for surgical treatment of nodules and the only risk factor for malignancy.

The rate of surgical treatment for indeterminate thyroid nodules that underwent MPT testing was only 24% when all patients in our study were considered. Surgery rates were artificially high at 45% in the study cohort, which was enriched for a higher number of patients who had surgical records compared to those who had existing clinical follow‐up records. A surgical treatment rate of 24%, as observed in our study, is in line with the rate required for a molecular test to be cost effective for the preoperative management of indeterminate thyroid nodules.^23^ Furthermore, the expected rate of surgery (11%) for a negative MPT diagnoses was consistent with the rate previously reported for benign cytology (Bethesda category II), supporting the utility of MPT as a rule out test.^24^ Our results also show that other risk factors for surgical treatment, such as nodule size and patient age, can also influence surgical decisions in such cases.

The performance of molecular tests for thyroid nodules has often been reported at a malignancy prevalence ranging from 24% to 32%, as most studies have only included patients who have undergone surgical treatment.^18^, ^19^, ^20^, ^25^ The rate of malignancy in our study cohort was only 14%, which is expected in a cohort of Bethesda category III and IV nodules that include patients who underwent conservative clinical follow up rather than surgery. Even at this lower rate of malignancy, patients with an MPT positive result only had a 43% probability of cancer‐free survival. In other words, patients with a positive MPT result had a 57% probability of malignancy, which supports the ability of a positive result to rule in the need for surgery. This probability is also similar to that which has been projected in a previous clinical validation study of MPT at a 14% malignancy prevalence.^18^ An MPT positive result placed patients at 13 times higher risk of malignancy compared to those with an MPT negative result, which carried a high probability (92%) of cancer‐free survival over 2 years follow‐up. This high probability of cancer‐free survival for negative results is consistent with a test that effectively rules out the need for surgery.

In our study, we examined the impact of reflex microRNA testing not only when nodules lacked mutations but also when nodules had weak driver mutations, given their association with both benign and malignant disease.^11^, ^12^, ^13^, ^14^, ^15^ We show that incorporation of microRNA testing of nodules with weak driver mutations into MPT significantly improved the rate of a negative MPT test result from 69% to 87% in our study cohort at 14% cancer prevalence. It ultimately identified high risk of cancer in a subset of patients with weak drivers when MPT results were positive and downgraded risk when MPT results were negative. As such, nodules with weak driver mutations and positive microRNA results can be considered high risk, warranting more aggressive surgical management options. Although downgrading malignancy risk did not statistically elevate the risk conferred by MPT negative results, it slightly reduced overall cancer‐free survival (87% vs 92%). Due to this result, we recommend that nodules with weak driver mutations and negative microRNA results be considered at moderate risk of cancer, where less aggressive management can be justified but cancer cannot be excluded. These risk classifications are consistent with those reported by others who have shown that microRNA analysis can help reclassify cancer risk of nodules with weak driver mutations.^16^

One impediment, we experienced in our study was the unavailability of clinical follow‐up records for some patients who underwent observation rather than surgery, which enriched our study cohort for patients who had surgical treatment for their nodule. This limitation is expected in real life clinical experience studies where patients with a negative molecular result are managed conservatively. This lack of records also reduced the rate of negative MPT results in the study cohort, given that most patients (93%) who were lost to follow up had a negative MPT result. Consistently, 80% of all patients (n = 337) in the study had negative MPT results compared to 69% in our study cohort (n = 180). In addition, patients who underwent clinical follow‐up only had a median follow‐up time of 9 months. Although the median time to diagnosis of malignancy was less than this (2 months), we cannot exclude the possibility that additional malignancies occurred after the minimum 3 month clinical follow‐up period. Furthermore, there were only a limited number of malignant cases in our study (n = 26).

Our multicenter clinical experience study supports results of others who have reported high positive and negative predictive value for MPT testing of indeterminate thyroid nodules.^18^ In our study, MPT results were predictive of real‐world decisions to surgically treat indeterminate thyroid nodules (Bethesda category III and IV). Importantly, those decisions were appropriately aligned with a patient's risk of malignancy over time based on MPT results. Patients with a negative MPT result had a high probability of survival without undergoing surgical treatment or having malignancy. An MPT positive result was a significant risk factor, independent of other factors, for both surgical treatment and for malignancy. Inclusion of microRNA testing of nodules with weak driver mutations into MPT can help identify high risk of cancer when microRNA results are positive and downgrade malignancy risk when microRNA results are negative. These results support the clinical utility of MPT testing in a real world clinical setting.

## AUTHOR CONTRIBUTIONS

JWS, AS, MF, RHB, JT, NF, PG, RG, and EG each participated in generating data for the study, reviewed data analysis and manuscript drafts, and approved the final draft of the manuscript.

## CONFLICT OF INTEREST

JWS, AS and RG are scientific consultants for Interpace Diagnostics.

## Supporting information


**Supplemental Figure S1** Sensitivity analysis for the expected rate of undergoing any type of surgical procedure (diagnostic lobectomy or total thyroidectomy) based on multiplatform mutation and microRNA (MPT) negative (green) or positive (red) results over various baseline rates of surgery. Dotted colored lines represent 95% confidence intervals. Solid vertical black lines represent the rate at which all patients in the study underwent surgery (24%) and the rate at which patients in the study cohort underwent surgery (45%).

**Supplemental Table S1** Univariate and multivariate hazard ratio (HR) analysis for risk of undergoing any type of surgical treatment (diagnostic lobectomy or total thyroidectomy) based on baseline patient characteristics and multiplatform mutation and microRNA testing (MPT).
**Supplemental Table S2**. Univariate and multivariate hazard ratio (HR) analysis for risk of malignancy based on baseline patient characteristics and multiplatform mutation and microRNA testing (MPT).
