# Supplementary figures and images for: Clinical impact of testing for mutations and microRNAs in thyroid nodules
Source: Diagn Cytopathol. 2019 Apr 23;47(8):758–64. doi: 10.1002/dc.24190 (PMC6766884; doi:10.1002/dc.24190)

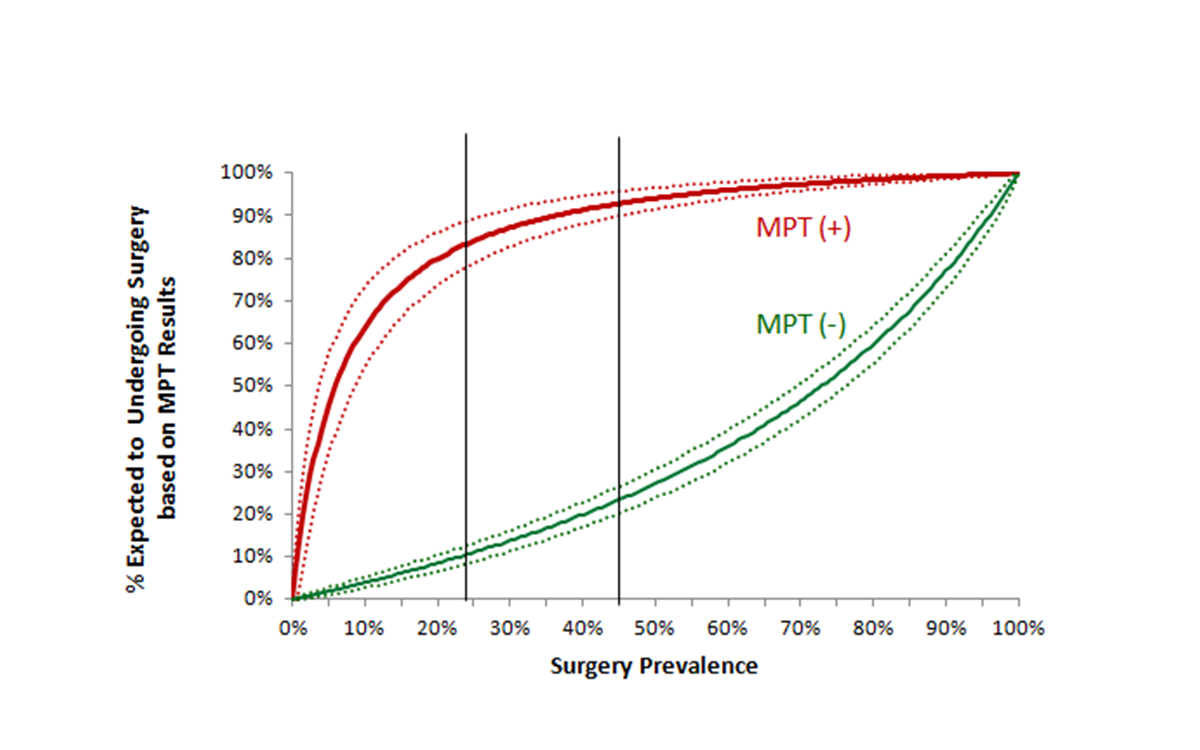

Supplement: Supplementary file 1 — Supplemental Figure S1 Sensitivity analysis for the expected rate of undergoing any type of surgical procedure (diagnostic lobectomy or total thyroidectomy) based on multiplatform mutation and microRNA (MPT) negative (green) or positive (red) results over various baseline rates of surgery. Dotted colored lines represent 95% confidence intervals. Solid vertical black lines represent the rate at which all patients in the study underwent surgery (24%) and the rate at which patients in the study cohort underwent surgery (45%). [file DC-47-758-s001.tif]
