# Supplementary material for: Clinical impact of testing for mutations and microRNAs in thyroid nodules
Source: Diagn Cytopathol. 2019 Apr 23;47(8):758–64. doi: 10.1002/dc.24190 (PMC6766884; doi:10.1002/dc.24190)
Supplement: Supplementary file 2 — Supplemental Table S1 Univariate and multivariate hazard ratio (HR) analysis for risk of undergoing any type of surgical treatment (diagnostic lobectomy or total thyroidectomy) based on baseline patient characteristics and multiplatform mutation and microRNA testing (MPT). Supplemental Table S2. Univariate and multivariate hazard ratio (HR) analysis for risk of malignancy based on baseline patient characteristics and multiplatform mutation and microRNA testing (MPT). [file DC-47-758-s002.docx]

**SUPPLEMENTAL TABLES**

Supplemental Table 1. Univariate and multivariate hazard ratio (HR) analysis for risk of undergoing any type of surgical treatment (diagnostic lobectomy or total thyroidectomy) based on baseline patient characteristics and multiplatform mutation and microRNA testing (MPT).

|  |  | **Univariate** | | | **Multivariate** | | |
| --- | --- | --- | --- | --- | --- | --- | --- |
| **Variable** | **Comparator Groups** | **HR** | **95% CI** | **P-value** | **HR** | **95% CI** | **P-value** |
| Bethesda category diagnosis (BCD) | BCD III vs. BCD IV | 0.74 | 0.45-1.21 | 0.2280 | 0.75 | 0.43-1.28 | 0.2972 |
| Age | Continuous | 0.98 | 0.96-0.99 | **0.0007** | 0.98 | 0.96-0.99 | **0.0103** |
| Sex | Female vs. Male | 0.96 | 0.59-1.55 | 0.8620 | 1.43 | 0.85-2.41 | 0.1795 |
| Family history of thyroid cancer | No vs. Yes | 1.56 | 0.78-3.13 | 0.2060 | 1.13 | 0.55-2.35 | 0.7351 |
| Family history of non-malignant thyroid disease | No vs. Yes | 1.32 | 0.83-2.10 | 0.2350 | 1.12 | 0.65-1.92 | 0.6729 |
| Personal history of non-thyroid cancer | No vs. Yes | 0.84 | 0.43-1.63 | 0.6110 | 0.84 | 0.41-1.73 | 0.6388 |
| Personal history of non-malignant thyroid disease | No vs. Yes | 1.19 | 0.77-1.84 | 0.4330 | 1.45 | 0.88-2.37 | 0.1411 |
| Nodule size | Continuous | 1.01 | 0.99-1.03 | 0.1560 | 1.03 | 1.01-1.05 | **0.0017** |
| MPT | Positive vs. Negative | 7.94 | 4.93-12.78 | **<0.0001** | 9.23 | 5.36-15.91 | **<0.0001** |

Supplemental Table 2. Univariate and multivariate hazard ratio (HR) analysis for risk of malignancy based on baseline patient characteristics and multiplatform mutation and microRNA testing (MPT).

|  |  | **Univariate** | | | **Multivariate** | | |
| --- | --- | --- | --- | --- | --- | --- | --- |
| **Variable** | **Comparator Groups** | **HR** | **95% CI** | **P-value** | **HR** | **95% CI** | **P-value** |
| Bethesda category diagnosis (BCD) | BCD III vs. BCD IV | 0.75 | 0.31-1.78 | 0.5090 | 0.72 | 0.27-1.89 | 0.4990 |
| Age | Continuous | 0.98 | 0.96-1.01 | 0.2250 | 1.00 | 0.97-1.03 | 0.8150 |
| Sex | Female vs Male | 1.45 | 0.66-3.20 | 0.3540 | 1.96 | 0.83-4.64 | 0.1250 |
| Family history of thyroid cancer | No vs Yes | 1.54 | 0.46-5.13 | 0.4840 | 1.06 | 0.30-3.70 | 0.9240 |
| Family history of non-malignant thyroid disease | No vs Yes | 0.95 | 0.40-2.26 | 0.9080 | 0.92 | 0.35-2.42 | 0.8630 |
| Personal history of non-thyroid cancer | No vs Yes | 0.49 | 0.11-2.06 | 0.3270 | 0.37 | 0.08-1.68 | 0.1970 |
| Personal history of non-malignant thyroid disease | No vs Yes | 1.14 | 0.53-2.46 | 0.7380 | 1.90 | 0.80-4.53 | 0.1480 |
| Nodule size | Continuous | 0.98 | 0.94-1.02 | 0.2700 | 0.99 | 0.95-1.03 | 0.6870 |
| MPT | Positive vs Negative | 11.1 | 4.49-27.63 | **<0.0001** | 13.36 | 4.79-37.22 | **<0.0001** |

**SUPPLEMENTAL FIGURE LEGEND**

Supplemental Figure 1. Sensitivity analysis for the expected rate of undergoing any type of surgical procedure (diagnostic lobectomy or total thyroidectomy) based on multiplatform mutation and microRNA (MPT) negative (green) or positive (red) results over various baseline rates of surgery. Dotted colored lines represent 95% confidence intervals. Solid vertical black lines represent the rate at which all patients in the study underwent surgery (24%) and the rate at which patients in the study cohort underwent surgery (45%).
